# Sensory afferent electrical stimulation to improve upper limb function in children with hemiparesis – a randomised controlled trial (SenseUp study protocol)

**DOI:** 10.3389/fped.2026.1886291

**Published:** 2026-08-12

**Authors:** Alisa Gschaidmeier, Kim Lory, Kevin Möri, André Moser, Tobias Nef, Kathleen Seidel, Cristina Simon-Martinez, Miriam Von Gunten, Jonathan Wermelinger, Roland Wiest, Regula Everts, Sebastian Grunt

**Affiliations:** 1 Division of Neuropaediatrics, Development and Rehabilitation, Department of Paediatrics, Inselspital, Bern University Hospital, University of Bern, Bern, Switzerland; 2 Graduate School for Health Sciences, University of Bern, Bern, Switzerland; 3 Gerontechnology and Rehabilitation Group, ARTORG Center for Biomedical Engineering Research, University of Bern, Bern, Switzerland; 4 Department of Clinical Research, University of Bern, Bern, Switzerland; 5 Department of Neurology, Inselspital, University Hospital Bern, University of Bern, Bern, Switzerland; 6 Department of Neurosurgery, Inselspital, Bern University Hospital, University of Bern, Bern, Switzerland; 7 Institute of Informatics, University of Applied Sciences Western Switzerland (HES-SO) Valais-Wallis, Sierre, Switzerland; 8 Department of Physiotherapy, Inselspital, Bern University Hospital, Bern, Switzerland; 9 Department of Diagnostic and Interventional Neuroradiology, Bern University Hospital, Inselspital, Bern, Switzerland

**Keywords:** accelerometry, functional networks, magnetic resonance imaging, motion tracking, neuroplasticity, pediatric stroke, transcranial magnetic stimulation, unilateral cerebral palsy

## Abstract

**Background:**

Children with hemiparesis present with sensory and motor deficits, which negatively affect quality of life. Sensory Afferent Electrical Stimulation (SAES) triggers action potentials in afferent nerve fibers, leading to increased sensorimotor afferent input. While proven effective in adults after stroke and safe in children with cerebral palsy through small studies, a systematic and large-scale investigation in the pediatric population is still missing. This protocol describes a study designed to investigate the efficacy and mechanisms of SAES.

**Methods:**

We will recruit 34 children and adolescents with spastic hemiparesis to participate in the prospective, single center, randomized controlled Bayesian phase II trial with a 5-week intervention period and a follow-up examination after 12 weeks. Participants will be randomly assigned to a 5-week SAES intervention or a control group consisting of treatment as usual. Before and after the SAES as well as at 12-week follow-up, clinical measures will be used to assess bimanual and unimanual hand functions (primary outcome: Assisting Hand Assessment AHA). Accelerometry and contactless motion tracking will be applied to evaluate everyday life upper limb functions. Neurophysiological methods such as structural and functional Magnetic Resonance Imaging and Transcranial Magnetic Stimulation will be performed to gain insight into neuroplastic mechanisms underlying SAES and are considered secondary outcomes.

**Discussion:**

Previous studies were limited by small cohorts and narrow outcome measures. Our study addresses these gaps while additionally investigating underlying neurophysiological mechanisms in children using MRI and TMS, providing a scientific basis for implementing such stimulation in practice.

**Clinical Trial Registration:**

clinicaltrials.gov, identifier (NCT06536634); kofam.ch, identifier (SNCTP000005950).

## 1 Background

Hemiparesis is characterized by motor impairments affecting one side of the body, often resulting in muscle stiffness, weakness, and reduced functional use of the affected limbs. In children, spastic hemiparesis can significantly impact motor development, daily activities, and quality of life (1, 2). Participation at home and in the community is related to hemiparesis severity (3). Early identification and efficient interventions are necessary to support functional independence and minimize functional constraints. Despite advances in therapeutic approaches, accurately assessing motor function and treatment outcomes remains challenging in young populations during development. Therefore, research is essential to refine diagnostic tools, enhance therapeutic strategies, and improve long-term outcomes for children with spastic hemiparesis.

Identification of efficient individual training methods for children with hemiparesis continues to present difficulties due to much variability in treatment response (4). In this study, we will investigate the effects and mechanisms of a special intervention, sensory afferent electrical stimulation (SAES). It is a type of peripheral stimulation that triggers action potentials in afferent nerve fibers, leading to increased sensory afferent input in the brain (5). In adult stroke patients with hemiparesis, SAES has been proven effective (6). Enhancement of sensory input, in the form of repetitive peripheral sensory electrical stimulation, can affect the excitability of the motor cortex and UL performance in adults (7). Small preliminary studies have shown improvements in motor and sensory function in children with hemiparesis using SAES (8), transcutaneous electrical nerve stimulation (9) and other types of sensory stimulation with a mesh glove (10) compared to the control group.

While SAES has shown promise in adults and small paediatric samples, previous studies were limited by small cohorts and a narrow range of outcome measures. Previous findings suggest that SAES is feasible in children with hemiparesis and may improve hand function and spasticity. However, the specific effects of SAES on sensory function, including tactile discrimination, stereognosis, and the integration of sensory input into motor performance, remain insufficiently explored. Additionally, the underlying neurophysiological mechanisms are still poorly understood. By using a multimodal assessment that combines clinical outcome measures with neurophysiological data, this trial aims to provide a more comprehensive understanding of potential intervention effects and mechanisms.

As a primary outcome, we will assess bimanual hand function with the Assisting Hand Assessment (AHA). The AHA assesses the quality of use of the affected hand during bimanual performance in individuals with hemiparesis.

To better understand the neuroplastic mechanisms, we will use a combination of neurophysiological technologies such as TMS and advanced neuroimaging techniques. The outcome after brain injury depends on the reorganization and compensatory potential of the brain. In rehabilitation medicine treatment strategies aim to influence these neuroplastic processes. Hence, we need a deeper understanding of the brain's capacity for reorganization after an injury.

TMS can probe the neurophysiology of the central nervous system by using strong magnetic fields to stimulate brain areas (11). In healthy adults, TMS showed that whole-hand SAES induces increased strength of corticospinal projections and intracortical changes such as decreased intracortical inhibition and increased intracortical facilitation, which may indicate long-term potentiation mechanisms (12, 13).

Resting state fMRI has been used to better understand motor ability of children with hemiparesis. Higher intrahemispheric connectivity was related to better motor outcomes in patients with hemiparesis (14). Aiming for a child-suited diagnostic, we use resting-state fMRI and arterial spin labelling (ASL) as task-free measurements to understand topographical connectivity at rest and cerebral blood flow patterns (15, 16). Utilizing ASL, one study demonstrated that arterial ischemic stroke (AIS) is associated to long-term alterations in cerebral blood flow in vital brain tissue, even in patients without hemiparesis. The extent of cerebral perfusion imbalance in children after AIS was found to be associated with their manual ability (16).

To better explore hand function, we will include contactless motion tracking and accelerometry, since the scoring of UL function might be experimenter-dependent and shows low sensitivity to treatment response (17).

Measurement tools that objectively quantify UL kinematics are useful to deepen the understanding of motor deficiencies and are often used to assess UL performance in children with CP (18, 19).

Camera-based motion tracking further extends the kinematic data by spatiotemporal parameters, which has the potential to identify clinically relevant movement patterns that may allow for improvements in patient specific therapies (20).

Previous work showed that inertial sensor measurements reliably identify paresis and correlate with clinical measurements; they can therefore provide a complementary dimension of assessment in clinical practice and during clinical trials aimed at improving UL function (21). Accelerometer data has been used to assess motor outcomes in children with CP undergoing constraint induced movement therapy (22), hand–arm bimanual intensive therapy (23), and action observation training (24). The data of the present study with children with hemiparesis will be used to better understand the disease related hand movement patterns.

The objective of the present study is to investigate whether SAES is more effective than the standard of treatment alone in children with hemiparesis. We will assess the efficacy of SAES using the AHA (primary endpoint) and novel clinical assessments such as kinematic evaluations and modern neurophysiological measures, namely structural MRI, resting-state functional MRI (rs-fMRI), arterial spin labelling (ASL) and transcranial magnetic stimulation (TMS) alongside contactless motion tracking and accelerometer data. This multimodal research approach will allow shedding light on the mechanisms underlying SAES, giving new insights into the neuroplastic properties of the developing brain.

## 2 Methods

### 2.1 Study design

**Figure 1:**
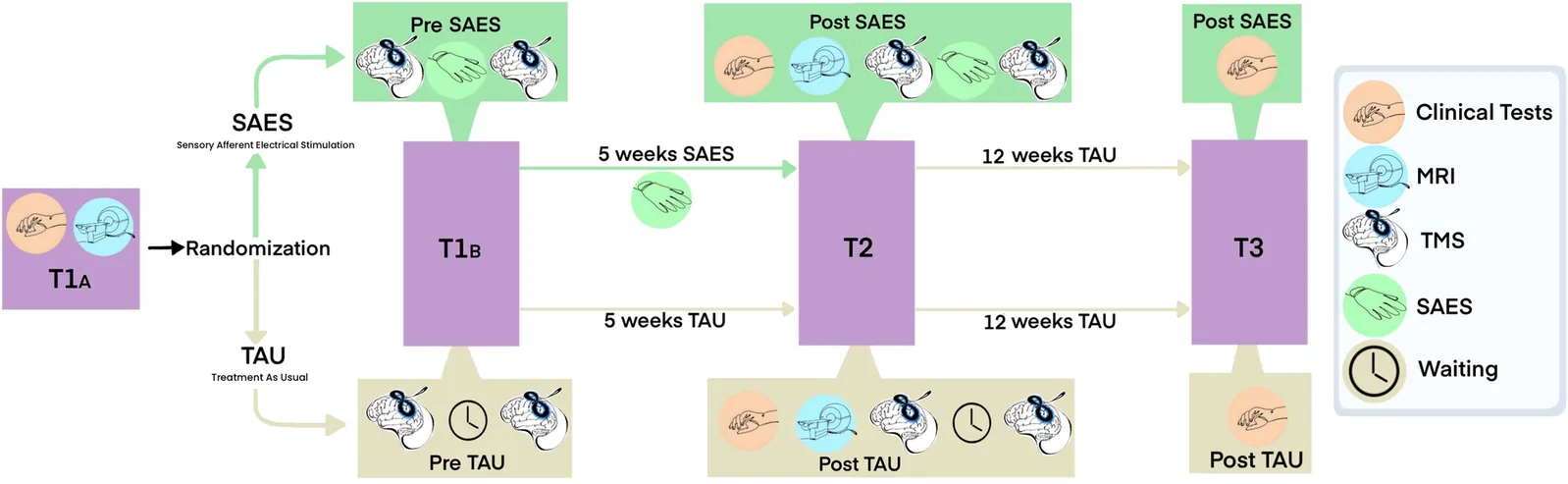
Study flow chart. T1A, first part of baseline assessment; T1B, second part of baseline assessment; T2, Assessment after intervention or 5-week waiting period; T3, follow-up assessment 12 weeks after intervention termination; TAU, Treatment as usual, SAES, sensory afferent electrical stimulation.

The SenseUp study is a prospective, single centre, randomized controlled Bayesian phase-II trial with a follow-up examination after 12 weeks (Figure 1). In total, thirty-four participants with hemiparesis will be included.

The assessments will take place at different sites at the University Hospital in Bern and at the Swiss Institute for Translational and Entrepreneurial Medicine. The trial protocol was developed in accordance with the SPIRIT guidelines.

### 2.2 Patients

Patients are eligible for the study if they have a diagnosis of hemiparesis with involvement of the UL following a unilateral brain lesion, are aged between 6 and 18 years, and diagnosed in a chronic state (>2 years since injury). Patients will be excluded due to the following reasons: psychiatric or medical conditions preventing the participation in the study or the training of the UL; botulinum toxin-injections in the last six months; trauma to the UL in the past year; hand surgery in the past two years; previous participation in a study investigating afferent electrical stimulation; performance of afferent stimulation therapy in the past six months; ongoing intensive training of the UL (therapy more than once a week in the last six months).

For MRI, participants will be excluded if they have implanted metal devices (e.g., braces, implants), an implanted shunt system, are pregnant or have claustrophobia. For TMS, the same criteria apply, with the addition of an exclusion in case of active epilepsy.

Informed consent will be obtained from a parent or legal guardian, and children over the age of fourteen will be asked to provide their own signed informed consent in addition.

### 2.3 Recruitment

Participants will be recruited via the University Children’s Hospital Bern, the Swiss CP Register, the Swiss Neuropediatric Stroke Registry, local specialists, neuropaediatricians in private practices and Swiss neuropaediatric centres.

### 2.4 Intervention

The experimental intervention will consist of SAES applied to the affected hand of the child in a home-based setting. Stimulation will be delivered using a Chattanooga Cefar® TENS dual-channel stimulator.

A stimulation glove electrode (STIMEX® Stimulation Glove, Pierenkemper GmbH, schwa-medico, Germany) will be applied to the affected hand and will serve as the anode. The glove consists of a silver-woven textile fabric designed to ensure homogeneous current distribution across the entire hand surface. To reduce skin impedance, the glove will be lightly moistened with water prior to application. The corresponding cathode will be positioned on the dorsal aspect of the proximal third of the forearm.

For the second stimulation channel, two self-adhesive electrodes (5 cm × 5 cm) will be placed on the volar aspect of the forearm over the wrist and finger flexor muscle group. The anode will be positioned at the mid-forearm level and the cathode proximally to it.

Stimulation will be delivered using continuous asymmetric biphasic rectangular pulses at a frequency of 40 Hz and a pulse width of 300 μs. Stimulation intensity will be individually titrated in increments of 0.5 mA until the participant first perceives a tingling sensation. The intensity will then be reduced by 0.5 mA and maintained below the individual sensory perception threshold for the remainder of the session. This subsensory stimulation approach has been selected to minimize conscious sensory perception and motor activation while aiming to modulate afferent neural pathways.

Intervention delivery will be combined with standard of care treatment. The first stimulation session will take place at the University Children's Hospital in Bern while subsequent sessions will be performed at home.

Before the home-based intervention, participants and their families will receive hands-on training during their first stimulation session, conducted by trained study team members. This training covers correct glove application and SAES device handling. A standardised instruction sheet with photographs and guiding steps will be provided for reference at home.

Adherence will be monitored indirectly via the MyCap application (REDCap extension, explained in 2.8.9. MyCap App), through which participants or their parents will be instructed to log each stimulation session. The study team will review logs regularly and contacts families when sessions are not recorded as expected. Missed sessions will not be rescheduled. Objective stimulation times will be verified post-intervention upon return of the SAES device, which logs actual stimulation duration internally.

Participants completing above or equal to 20 out of 25 sessions (80%) are classified as compliant.

### 2.5 Control intervention

The control intervention (treatment as usual, TAU) consists of the standard of care treatment, such as the prescribed conventional occupational therapy or physiotherapy.

### 2.6 Randomisation

The group allocation will follow a stratified randomization in a 1:1 ratio to ensure well-balanced division of the whole study population. The implemented stratification factors include age (6–12 years vs. 12–18 years) and the baseline measurement for the AHA, conducted at the first study visit. The randomization sequence is implemented in REDCap and generated by an independent data manager at the Clinical Trial Unit (CTU) Bern.

### 2.7 Primary outcome

The primary endpoint is bimanual hand function after treatment, measured with AHA at week 5. The AHA assesses the quality of use of the affected hand during bimanual performance in individuals with hemiparesis. The Kids-AHA has been validated for children with Hemiparesis aged 18 months to 12 years and has proven reliability and sensitivity to monitor changes over time (25, 26). Similarly, the Ad-AHA (version for adolescents) has shown good-to-excellent interrater and test–retest reliability (27). The AHA scoring criteria remain the same for both children and adolescents, while the test activities differ to accommodate the different age groups (26). The raw sum scores (0–80 points) are converted to AHA-units (interval logit measures) ranging from 0 to 100 (28). Higher AHA units indicate better bimanual performance. A change of five units in the logit-based AHA scale within-participant change has been described to lead to a clinically meaningful difference. However, it still remains under discussion and requires further investigation, as even small changes can be significant and meaningful for an individual, if they imply functional gains (29). The post-intervention measurement and the 12-week follow-up will be evaluated by a blinded assessor who is not otherwise involved in the trial.

### 2.8 Secondary outcomes

#### 2.8.1 Clinical measurements

For unimanual performances, maximal palmar grasp strength will be assessed using a pneumatic dynamometer (Baseline®, USA). Hand function will be classified through the Manual Ability Classification System (30). To assess hand and wrist positioning, two established scoring systems will be employed: the Zancolli classification for evaluating deformities commonly observed in cerebral palsy and the House classification for the thumb-in-palm deformity (31). The quality of unilateral UL movement will be evaluated using the Jebson Taylor Hand Function test, which measures unimanual fine and gross hand function using stimulated activities of daily life (32). Mirror movements will be assessed via the Woods and Teuber Scale with excellent reliability for scoring mirror movements (33). For measurements of sensory functions*,* the Semmes-Weinstein monofilaments will be administered (34). For assessment of stereognosis of the affected hand, the Jamar toolkit will be used to evaluate the unimanual tactile object recognition. To measure the bimanual hand interaction in daily tasks, a shortened, age- and patient-population-adapted version of the Chedoke Arm and Hand Activity Inventory (CAHAI) will be performed (35).

#### 2.8.2 Questionnaires

Two Questionnaires will be completed. Hand function during daily activities will be assessed by the Children's Hand-use Experience Questionnaire CHEQ (36) and the health-related quality of life will be evaluated with the KIDSCREEN-27 (37).

Occupational performance priorities will be assessed using the Canadian Occupational Performance Measure (COPM) (38) a semi-structured, client-centered interview that captures perceived performance and satisfaction. It is widely used in children with cerebral palsy to evaluate goal achievement and is sensitive to clinically meaningful change (39).

#### 2.8.3 Magnetic resonance imaging (MRI)

Magnetic resonance imaging (MRI) of the brain will be conducted in a 3-T scanner at the Institute of Diagnostic and Interventional Neuroradiology, University Hospital Inselspital, Bern. A high-resolution T1-weighted Magnetization-Prepared Rapid Gradient Echo (MPRAGE) sequence will be used to assess grey matter thickness, volume and surface area (TR = 1,950.0 ms, TE = 2.26 ms, TI = 900 ms, Flip Angle = 9°, FoV = 256 mm, voxel size = 1.0 × 1.0 × 1.0 mm). Furthermore, T2-weighted Fluid-Attenuated Inversion Recovery (FLAIR) sequence will be acquired (TR = 5,000 ms, TE = 388 ms, TI = 1,800 ms, FoV = 256 mm, voxel size = 0.5 × 0.5 × 1.0 mm). The FLAIR will help to better assess the cerebral lesion volume and location especially close to the cerebrospinal fluid (CSF) (40).

Resting-state functional MRI (rsfMRI) will be performed to assess the spontaneous fluctuations in blood oxygen level dependent (BOLD) signal. The rsfMRI will be performed using a multiband echo-planar imaging (EPI) sequence (TR = 1,950.0 ms, TE = 2.26 ms, TI = 900 ms, Flip Angle = 9°, FoV = 256 mm, voxel size = 1.0 × 1.0 × 1.0 mm) (41). Arterial spin labelling (ASL) will be performed to assess blood flow imbalances and alterations using a 3D background-suppressed pseudo-continuous arterial spin labeling (3D BS pcASL) sequence [TR = 4,200 ms, TE = 21.08 ms, FoV = 240 mm, voxel size = 3.8 × 3.8 × 3.0 mm, Labeling duration = 1,800 ms, Post-labeling delay (PLD) = 2000ms]. 3D BS pcASL is the sequence currently recommended by the International Society for Magnetic Resonance in Medicine (ISMRM) for research purposes (42). The MRI scans will last 30 min.

#### 2.8.4 Transcranial magnetic stimulation (TMS)

TMS is an intervention with minimal risk, excellent tolerability and increasingly sophisticated ability to interrogate neurophysiology and plasticity in pediatric populations (43). TMS will be delivered using a Nexstim NBS System 5 (220-240 V, 50/60 Hz, 600 VA) with a figure-of-eight coil and MRI-guided neuronavigation (nTMS). To enable the neuronavigation, structural MPRAGE MRI images of the participant will be used. During the nTMS session, muscle activity will be continuously monitored using electromyography (EMG) with surface electrodes (Ambu Neuroline) over the targeted muscle (M. abductor pollicis longus). Resting motor threshold (RMT) was defined as the lowest stimulation eliciting an MEP ≥50 µV in the target muscle. The presumed hot spot of arm muscles will be determined as described before (44) by functional mapping after searching the area around the anatomical landmarks (omega sign of precentral gyrus for the hand muscles). Stimulation will be administered to the motor cortex using two figure-of-8 coils, delivering monophasic pulses via the Nexstim system connected to a Magstim 200^2^ (the Magstim Company Limited, Whitland, UK) via a trigger box. Single pulse recruitment courves were obtained at 110%–150% RMT (whenever well tolerated by the child). Both hemispheres will be stimulated simultaneously to assess interhemispheric inhibition in both directions, lesion to non-lesion and non-lesion to lesion. The interhemispheric inhibition was assessed using a paired-pulse paradigm with a conditioning stimulaus at 80% RMT and a test stimulus at 120% RMT, at interstimulus intervals of 10 ms and 40 ms, corresponding to short, and long-latency interhemispheric inhibition respectively. The TMS measurements will be conducted at the baseline measurement and at the post-intervention measurement for the participants eligible and consenting to the TMS.

#### 2.8.5 Accelerometer data

UL activity in daily life will be monitored using acceleration data from an Axivity AX6 (Axivity Ltd, Newcastle, United Kingdom) data logger. All participants will be equipped with one data logger attached to each wrist. These logging devices will be secured using custom designed bracelets made from thick elastic bands and Velcro. The devices operate in an Inertial Measurement Unit (IMU) mode at a sampling rate of 100 Hz. Specifications include a measurement range of ±8 g for acceleration and ±500°/s for angular velocity, with a 16-bit resolution for both the accelerometer and gyroscope. Both devices will be given to each participant at 4 time points for individuals in the intervention group and at 3 time points for participants in the control group. At each measurement time point, the devices will be worn on both wrists for 48 consecutive hours in the child's day-to-day life.

#### 2.8.6 Motion tracking

The NeuroTec-Loft is equipped with various sensor systems that monitor and assess movement abilities of individuals with neurological disorders while they perform activities of daily living (ADL).

The UL movements of the participants are recorded by contactless motion tracking systems using two different RGB-based multi-camera systems. The first system comprises six XIMEA xiX (XIMEA GmbH, Germany) RGB cameras with a resolution of 3.1 MP and a frame rate of 90 fps. The second system uses six Miqus Hybrid (Qualisys AB, Göteborg, Sweden) motion tracking cameras in RGB mode with a resolution of 2 MP and a frame rate of 85 fps. To retrieve quantitative movement data, trained UL pose estimation models are applied to each of the captured recordings. The tracked anatomical landmarks return the Cartesian coordinates allowing a reconstruction of three-dimensional skeletal models that provide kinematic and spatiotemporal parameters such as acceleration, velocity, joint angles, and positional information of body segments.

#### 2.8.7 Pain

Participants will report their pain levels before and after each SAES training session on the MyCap application using a five-point-Likert scale. The primary function of this is to give patients and their families a low threshold option of reporting discomfort.

#### 2.8.8 Sociodemographic variables

Age, gender, socioeconomic status, symptoms at manifestation, if available disease-specific outcome measurements (e.g., a brief medical history, detailed disease-specific outcome measures) will be assessed. Medical background variables will be recorded, including: current or past medical treatments and therapies (such as physical therapy, occupational therapy, neuropsychological therapy), medication, epileptic seizures, time of lesion, time since lesion, severity of the hemiparesis, comorbidities and general medical records regarding the hemiparesis.

#### 2.8.9 Mycap app

MyCap, an extension of REDCap™, is a flexible, participant-facing mobile application designed to facilitate survey data collection and active tasks in research studies (45). The MyCap app will serve as the primary platform for participant engagement and monitoring during the five-week stimulation at home.

### 2.9 Adverse events

(Serious) Adverse Events [(S)AE] will be classified according to the Common Terminology Criteria for Adverse Events (CTCAE).

### 2.10 Sample size

We calculated that 34 patients are needed to claim a successful trial with a simulated power of 84.3% under an assumed standardized effect size of 0.6 from 5,000 Monte-Carlo simulations. We used an informative Gaussian prior with a mean of 1 and a standard deviation of 1 for the expected standardized effect size. As no AHA-based effect size related to peripheral electrical stimulation was available in the literature, this prior was informed by previously reported effects in a similar population for the Jebsen–Taylor Hand Function test reporting a standardised effect size of 1.97 (9) but shifted towards a more conservative effect size. We defined a posterior probability threshold of 90% as trial success.

### 2.11 Statistical analyses

The primary endpoint will be analysed using a Bayesian linear regression model with the above-specified Gaussian informative prior with mean of 1 and standard deviation of 1. We claim trial success if the probability that baseline-adjusted posterior mean difference of the primary endpoint between the groups at week 5 is greater than zero is greater than 90%. We report the posterior treatment allocation mean difference with 90% credible intervals as effect measure. Regression models are adjusted for baseline AHA and age. We perform the following sensitivity analyses. First, we use a non-informative centered Gaussian prior with variance 1,000 for the effect size. Second, because the logit-based AHA score is bounded between 0 and 100 we use Bayesian beta regression model with a logit link for the mean parameter and an identity link on the precision parameter. Because the effect estimate from a beta regression is on a log-odds scale we use a non-informative centered Gaussian distribution with variance 1,000 as prior for the effect size. We report marginal mean differences with 90% credible intervals to allow a direct comparison of the effect measure with the primary analysis.

Secondary outcomes will be descriptively reported over time (post-treatment and 12-week follow-up) and treatment group. Subgroup analyses by gender and MACS levels were *a priori* specified. We will assess the proportion of missing values only in variables, which affect the interpretation of the estimands for the primary outcome. A detailed analysis strategy (including sensitivity and subgroup analysis) has been formulated in a Statistical Analysis Plan (SAP), which was finalised before one-third of the patients were recruited. Missing data will be multiply imputed in case the proportion of missingness exceeds 5%. Otherwise, we replace missing values by the median within the corresponding treatment group. The proportion of missing values in the primary outcome at week 5 is assessed before database lock and start of the analysis. At this time point the imputation model and specification is detailed in an SAP amendment. For secondary outcomes and missing baseline variables we report complete case results.

### 2.12 Ethics and dissemination

The study was reviewed by the CTU in Bern and was approved by the ethics committee of the canton of Bern, Switzerland (2024-00982, accepted on the 21.05.2024, the latest Amendment 2, Version 1.3 was accepted on 11.06.2025). The manuscript includes all revisions. In case of future amendments, all relevant parties will be adequately informed.

Official monitoring by the CTU Bern was conducted over the course of the study.

Data will be securely stored using REDCap, hosted at CTU Bern. The corresponding data management file is available from the authors upon reasonable request.

The study is conducted according to the principles of the Declaration of Helsinki in its latest form.

Data privacy is ensured; each participant will receive a unique, de-identified study code, securely stored in a password-protected file.

Results will be published in peer-reviewed journals and presented at scientific meetings. Authorship will follow International Committee of Medical Journal Editors (Vancouver Convention) guidelines.

## 3 Discussion

Hemiparesis is one of the leading causes of childhood disability, with a negative impact on participation and everyday life. Despite advances in therapeutic approaches, accurately assessing motor function and treatment outcomes remains challenging in young populations during development. SAES has been proven effective in adult populations. Due to developmental differences in children and adults, it is crucial to study the effect of SAES in the paediatric population independently of the adult population.

This study will examine the efficacy of SAES to improve UL motor and sensory function and activity in everyday life. We will use innovative, novel methods to assess bimanual hand functions and a multimodal research approach to disentangle the neuroplastic mechanisms underlying SAES. Our primary outcome, the AHA, was selected due to its clinical relevance and because it reflects spontaneous use of the affected hand in bimanual activities, which is highly relevant to everyday functioning. Secondary endpoints assess motor and sensory functions to explore potential mechanisms of action and determine whether improved hand use is linked to changes in motor control and sensorimotor integration, offering further insight into clinical outcomes. Our study focuses on treatment optimization, as our findings will give insights into how SAES impacts hand functions and cerebral sensorimotor networks in a comparably large patient cohort. Hence, the present study has high clinical implications and will contribute to the enhancement of new diagnostic treatment strategies.

## 4 Limitations

This study design has several limitations we would like to acknowledge. Firstly, the relatively small sample size of 17 children per study arm was calculated having the primary outcome in mind, which poses considerable limitations on the statistical power of our secondary outcomes. This limitation is most pronounced for the MRI and TMS outcomes, since these two assessments impose additional exclusion criteria further reducing the number of participants. A second limitation concerns the sample size calculation itself. As no AHA-based effect sizes were available from prior sensory afferent electrical stimulation trials in this population, the informative prior was derived from an effect size for a different outcome measure in a related electrostimulation trial (9). While this effect size was scaled down conservatively, this introduces uncertainty into the sample size and power calculation.

A third limitation concerns the missing blinding of participants and their families, which is not feasible in the design of this intervention trial. Since participants are aware whether or not they are receiving the stimulation, this could potentially bias our outcome measures. This is especially true for our subjective outcomes such as the KidScreen or the COPM.

## References

[1] Gilson KM Davis E Reddihough D Graham K Waters E. Quality of life in children with cerebral palsy: implications for practice. J Child Neurol. (2014) 29(8):1134–40. 10.1177/0883073814535502 24870369

[2] Vila-Nova F Dos Santos Cardoso de Sá C Oliveira R Cordovil R. Differences in leisure physical activity participation in children with typical development and cerebral palsy. Dev Neurorehabil. (2021) 24(3):180–6. 10.1080/17518423.2020.1819461 32981411

[3] Simon-Martinez C Kamal S Frickmann F Steiner L Slavova N Everts R. Participation after childhood stroke: is there a relationship with lesion size, motor function and manual ability? Eur J Paediatr Neurol. (2021) 35:16–26. 10.1016/j.ejpn.2021.09.010 34592642

[4] Novak I Honan I. Effectiveness of paediatric occupational therapy for children with disabilities: a systematic review. Aust Occup Ther J. (2019) 66(3):258–73. 10.1111/1440-1630.12573 30968419 PMC6850210

[5] Peurala S Pitkänen K Sivenius J Tarkka I. Cutaneous electrical stimulation may enhance sensorimotor recovery in chronic stroke. Clin Rehabil. (2002) 16(7):709–16. 10.1191/0269215502cr543oa 12428819

[6] Golaszewski SM Bergmann J Christova M Kunz AB Kronbichler M Rafolt D. Modulation of motor cortex excitability by different levels of whole-hand afferent electrical stimulation. Clin Neurophysiol. (2012) 123(1):193–9. 10.1016/j.clinph.2011.06.010 21764634

[7] Krieg SM Lioumis P Mäkelä JP Wilenius J Karhu J Hannula H. Protocol for motor and language mapping by navigated TMS in patients and healthy volunteers; workshop report. Acta Neurochir (Wien). (2017) 159(7):1187–95. 10.1007/s00701-017-3187-z 28456870

[8] Mäenpää H Jaakkola R Sandström M Airi T Wendt L. Electrostimulation at sensory level improves function of the upper extremities in children with cerebral palsy: a pilot study. Dev Med Child Neurol. (2004) 46(2):84–90. 10.1111/j.1469-8749.2004.tb00456.x 14974632

[9] Alhusaini AA Fallatah S Melam GR Buragadda S. Efficacy of transcutaneous electrical nerve stimulation combined with therapeutic exercise on hand function in children with hemiplegic cerebral palsy. Somatosens Mot Res. (2019) 36(1):49–55. 10.1080/08990220.2019.1584555 30913943

[10] Azzam AM. Efficacy of mesh glove sensory stimulation on spasticity control in hemiplegic CP. Indian J Physiother Occup Ther. (2012) 19:19–23.

[11] Di Lazzaro V Ziemann U Lemon RN. State of the art: physiology of transcranial motor cortex stimulation. Brain Stimul. (2008) 1(4):345–62. 10.1016/j.brs.2008.07.004 20633393

[12] Golaszewski S Kremser C Wagner M Felber S Aichner F Dimitrijevic MM. Functional magnetic resonance imaging of the human motor cortex before and after whole-hand afferent electrical stimulation. Scand J Rehabil Med. (1999) 31(3):165–73. 10.1080/003655099444506 10458314

[13] Golaszewski SM Siedentopf CM Koppelstaetter F Rhomberg P Guendisch GM Schlager A. Modulatory effects on human sensorimotor cortex by whole-hand afferent electrical stimulation. Neurology. (2004) 62(12):2262–9. 10.1212/WNL.62.12.2262 15210892

[14] Steiner L Homan S Everts R Federspiel A Kamal S Rodriguez JAD. Functional connectivity and upper limb function in patients after pediatric arterial ischemic stroke with contralateral corticospinal tract wiring. Sci Rep. (2021) 11(1):5490. 10.1038/s41598-021-84671-2 33750854 PMC7943570

[15] Simon-Martinez C Jaspers E Alaerts K Ortibus E Balsters J Mailleux L. Influence of the corticospinal tract wiring pattern on sensorimotor functional connectivity and clinical correlates of upper limb function in unilateral cerebral palsy. Sci Rep. (2019) 9(1):8230. 10.1038/s41598-019-44728-9 31160679 PMC6547689

[16] Leistner R Everts R Federspiel A Kornfeld S Slavova N Steiner L. Cerebral blood flow imbalance is associated with motor outcome after pediatric arterial ischemic stroke. PLoS One. (2019) 14(10):e0223584. 10.1371/journal.pone.0223584 31603919 PMC6788710

[17] Mailleux L Jaspers E Ortibus E Simon-Martinez C Desloovere K Molenaers G. Clinical assessment and three-dimensional movement analysis: an integrated approach for upper limb evaluation in children with unilateral cerebral palsy. PLoS One. (2017) 12(7):e0180196. 10.1371/journal.pone.0180196 28671953 PMC5495347

[18] Francisco-Martínez C Prado-Olivarez J Padilla-Medina JA Díaz-Carmona J Pérez-Pinal FJ Barranco-Gutiérrez AI. Upper limb movement measurement systems for cerebral palsy: a systematic literature review. Sensors (Basel). (2021) 21(23):7884. 10.3390/s21237884 34883885 PMC8659477

[19] Jaspers E Desloovere K Bruyninckx H Molenaers G Klingels K Feys H. Review of quantitative measurements of upper limb movements in hemiplegic cerebral palsy. Gait Posture. (2009) 30(4):395–404. 10.1016/j.gaitpost.2009.07.110 19679479

[20] Jaspers E Desloovere K Bruyninckx H Klingels K Molenaers G Aertbeliën E. Three-dimensional upper limb movement characteristics in children with hemiplegic cerebral palsy and typically developing children. Res Dev Disabil. (2011) 32(6):2283–94. 10.1016/j.ridd.2011.07.038 21862283

[21] Newman CJ Bruchez R Roches S Jequier Gygax M Duc C Dadashi F. Measuring upper limb function in children with hemiparesis with 3D inertial sensors. Childs Nerv Syst. (2017) 33(12):2159–68. 10.1007/s00381-017-3580-1 28842792

[22] Goodwin BM Sabelhaus EK Pan Y-C Bjornson KF Pham KLD Walker WO. Accelerometer measurements indicate that arm movements of children with cerebral palsy do not increase after constraint-induced movement therapy (CIMT). Am J Occup Ther. (2020) 74(5):7405205100p1–p9. 10.5014/ajot.2020.040246 32804628 PMC7430726

[23] Gardas SS Lysaght C McMillan AG Kantak S Willson JD Patterson CG. Bimanual movement characteristics and real-world performance following hand–arm bimanual intensive therapy in children with unilateral cerebral palsy. Behav Sci. (2023) 13(8):681. 10.3390/bs13080681 37622821 PMC10451828

[24] von Gunten M Botros A Tobler-Ammann B Nef T Grunt S. Action observation training to improve upper limb function in infants with unilateral brain lesion–a feasibility study. Dev Neurorehabil. (2023) 26(4):234–43. 10.1080/17518423.2023.2193630 36949659

[25] Krumlinde-Sundholm L Holmefur M Kottorp A Eliasson A-C. The assisting hand assessment: current evidence of validity, reliability, and responsiveness to change. Dev Med Child Neurol. (2007) 49(4):259–64. 10.1111/j.1469-8749.2007.00259.x 17376135

[26] Louwers A Beelen A Holmefur M Krumlinde-Sundholm L. Development of the assisting hand assessment for adolescents (Ad-AHA) and validation of the AHA from 18 months to 18 years. Dev Med Child Neurol. (2016) 58(12):1303–9. 10.1111/dmcn.13168 27291981

[27] Louwers A Krumlinde-Sundholm L Boeschoten K Beelen A. Reliability of the assisting hand assessment in adolescents. Dev Med Child Neurol. (2017) 59(9):926–32. 10.1111/dmcn.13465 28555755

[28] Holmefur MM Krumlinde-Sundholm L. Psychometric properties of a revised version of the assisting hand assessment (Kids-AHA 5.0). Dev Med Child Neurol. (2016) 58(6):618–24. 10.1111/dmcn.12939 26507383

[29] Krumlinde-Sundholm L. Reporting outcomes of the assisting hand assessment: what scale should be used? Dev Med Child Neurol. (2012) 54(9):807–8. 10.1111/j.1469-8749.2012.04361.x 22803624

[30] Eliasson A-C Krumlinde-Sundholm L Rösblad B Beckung E Arner M Öhrvall A-M. The manual ability classification system (MACS) for children with cerebral palsy: scale development and evidence of validity and reliability. Dev Med Child Neurol. (2006) 48(7):549–54. 10.1017/S0012162206001162 16780622

[31] Mcconell K Johnston L Kerr C. Upper limb function and deformity in cerebral palsy: a review of classification systems. Dev Med Child Neurol. (2011) 53(9):799–805. 10.1111/j.1469-8749.2011.03953.x 21434888

[32] Jebsen RH Taylor N Trieschmann RB Trotter MJ Howard LA. An objective and standardized test of hand function. Arch Phys Med Rehabil. (1969) 50(6):311–9. 5788487

[33] Magne VA Adde L Hoare B Klingels K Simon-Martinez C Mailleux L. Assessment of mirror movements in children and adolescents with unilateral cerebral palsy: reliability of the Woods and Teuber scale. Dev Med Child Neurol. (2021) 63(6):736–42. 10.1111/dmcn.14806 33469938

[34] Bell-Krotoski JA Fess EE Figarola JH Hiltz D. Threshold detection and Semmes-Weinstein monofilaments. J Hand Ther. (1995) 8(2):155–62. 10.1016/S0894-1130(12)80314-0 7550627

[35] Barreca SR Stratford PW Lambert CL Masters LM Streiner DL. Test-retest reliability, validity, and sensitivity of the Chedoke arm and hand activity inventory: a new measure of upper-limb function for survivors of stroke. Arch Phys Med Rehabil. (2005) 86(8):1616–22. 10.1016/j.apmr.2005.03.017 16084816

[36] Amer A Eliasson AC Peny-Dahlstrand M Hermansson L. Validity and test-retest reliability of Children’s Hand-use Experience Questionnaire in children with unilateral cerebral palsy. Dev Med Child Neurol. (2016) 58(7):743–9. 10.1111/dmcn.12991 26610725

[37] Robitail S Ravens-Sieberer U Simeoni M-C Rajmil L Bruil J Power M. Testing the structural and cross-cultural validity of the KIDSCREEN-27 quality of life questionnaire. Qual Life Res. (2007) 16:1335–45. 10.1007/s11136-007-9241-1 17668291

[38] McColl MA Law M Baptiste S Pollock N Carswell A Polatajko HJ. Targeted applications of the Canadian occupational performance measure. Can J Occup Ther. (2005) 72(5):298–300. 10.1177/000841740507200506 16435590

[39] Sakzewski L Boyd R Ziviani J. Clinimetric properties of participation measures for 5- to 13-year-old children with cerebral palsy: a systematic review. Dev Med Child Neurol. (2007) 49(3):232–40. 10.1111/j.1469-8749.2007.00232.x 17355482

[40] Bakshi R Ariyaratana S Benedict RH Jacobs L. Fluid-attenuated inversion recovery magnetic resonance imaging detects cortical and juxtacortical multiple sclerosis lesions. Arch Neurol. (2001) 58(5):742–8. 10.1001/archneur.58.5.742 11346369

[41] Feinberg DA Moeller S Smith SM Auerbach E Ramanna S Glasser MF. Multiplexed echo planar imaging for sub-second whole brain FMRI and fast diffusion imaging. PLoS One. (2010) 5(12):e15710. 10.1371/journal.pone.0015710 21187930 PMC3004955

[42] Alsop DC Detre JA Golay X Günther M Hendrikse J Hernandez-Garcia L. Recommended implementation of arterial spin-labeled perfusion MRI for clinical applications: a consensus of the ISMRM perfusion study group and the European consortium for ASL in dementia. Magn Reson Med. (2015) 73(1):102–16. 10.1002/mrm.25197 24715426 PMC4190138

[43] Rajapakse T Kirton A. non-invasive brain stimulation in children: applications and future directions. Transl Neurosci. (2013) 4(2):217–33. 10.2478/s13380-013-0116-3 PMC3807696 24163755

[44] Seidel K Häni L Lutz K Zbinden C Redmann A Consuegra A. Postoperative navigated transcranial magnetic stimulation to predict motor recovery after surgery of tumors in motor eloquent areas. Clin Neurophysiol. (2019) 130(6):952–9. 10.1016/j.clinph.2019.03.015 30981901

[45] Harris PA Swafford J Serdoz ES Eidenmuller J Delacqua G Jagtap V. MyCap: a flexible and configurable platform for mobilizing the participant voice. JAMIA open. (2022) 5(2):ooac047. 10.1093/jamiaopen/ooac047 35673353 PMC9165428

